# Wnts in adult brain: from synaptic plasticity to cognitive deficiencies

**DOI:** 10.3389/fncel.2013.00224

**Published:** 2013-12-03

**Authors:** Carolina A. Oliva, Jessica Y. Vargas, Nibaldo C. Inestrosa

**Affiliations:** ^1^Centro de Envejecimiento y Regeneración, Facultad de Ciencias Biológicas, Pontificia Universidad Católica de ChileSantiago, Chile; ^2^Departamento de Biologïa Celular y Molecular, Facultad de Ciencias Biológicas, Pontificia Universidad Católica de ChileSantiago, Chile

**Keywords:** Wnt in adult brain, neurodegenerative diseases, Wnt in circuit development, Wnt signaling pathways, spontaneous activity, activity-dependent processes

## Abstract

During development of the central nervous system the Wnt signaling pathway has been implicated in a wide spectrum of physiological processes, including neuronal connectivity and synapse formation. Wnt proteins and components of the Wnt pathway are expressed in the brain since early development to the adult life, however, little is known about its role in mature synapses. Here, we review evidences indicating that Wnt proteins participate in the remodeling of pre- and post-synaptic regions, thus modulating synaptic function. We include the most recent data in the literature showing that Wnts are constantly released in the brain to maintain the basal neural activity. Also, we review the evidences that involve components of the Wnt pathway in the development of neurological and mental disorders, including a special emphasis on *in vivo *studies that relate behavioral abnormalities to deficiencies in Wnt signaling. Finally, we include the evidences that support a neuroprotective role of Wnt proteins in Alzheimer’s disease. We postulate that deregulation in Wnt signaling might have a fundamental role in the origin of neurological diseases, by altering the synaptic function at stages where the phenotype is not yet established but when the cognitive decline starts.

## INTRODUCTION

Among all developmental processes that involve Wnt signaling, some of the more prominent are the primary embryo axis formation, segmentation processes, and appendage patterning in *Drosophila*; organogenesis from worms to mice, as well as stem cell proliferation (Niehrs, 2010; Jackson and Eisenmann, 2012; Wang et al., 2012; Wray and Hartmann, 2012). Wnt signaling has been implicated in various diseases, including colon cancer and melanoma (Polakis, 2012), and also in neurodegenerative diseases (Inestrosa and Arenas, 2010; Berwick and Harvey, 2012), reflecting its relevance in fundamental biological processes across species (Clevers and Nusse, 2012). Because *Wnt* gene is fundamental to determine a normal neural phenotype (McMahon and Moon, 1989b), in this review we focus on its function related to the central nervous system (CNS), from embryonic neural development to higher brain function in the adult brain.

The Wnt signaling comprises a complex cascade of components that are under many regulatory steps. The Wnt proteins family includes 19 members present in mammals. The prototypical Wnt receptor is the seven transmembrane-receptor Frizzled (Fz; Bhanot et al., 1996; Rulifson et al., 2000; van Amerongen et al., 2008). There are also co-receptors been described, such as the members of the low-density lipoprotein receptor-related protein 5/6 (LRP5/6; Tamai et al., 2000; Wehrli et al., 2000; Mao et al., 2001; MacDonald et al., 2009), the single-pass transmembrane receptors Tyr kinase-like orphan receptor (ROR), protein Tyr kinase 7 (PTK7), receptor Tyr kinase (RYK), and muscle skeletal receptor Tyr kinase (MUSK) (Oishi et al., 2003; Lu et al., 2004; Cadigan and Liu, 2006; Gordon and Nusse, 2006; Green et al., 2008; Jing et al., 2009; Fradkin et al., 2010; Minami et al., 2010; Peradziryi et al., 2012), and the co-receptors from the proteoglycan families (Kikuchi et al., 2011). There are several regulatory steps for the activity of these receptors. Not only they can be intracellularly phosphorylated, but also there are several secreted antagonists that can act extracellularly to modify their activity, like Cerberus and Dickkopf-related protein 1 (Dkk-1) that bind LRP blocking the interaction to Wnt/Frizzled (Mao et al., 2001); the secreted Frizzled-related protein (sFRP) that binds directly to Wnt because of the similarity they have with Fz (Finch et al., 1997; Rattner et al., 1997); the Wnt inhibitory factor (WIF); Sclerostin (and its homolog Wise; Cruciat and Niehrs, 2013), and two classes of Wnt agonists, the R-spondin family and Norrin (Cruciat and Niehrs, 2013).

**FIGURE 1:**
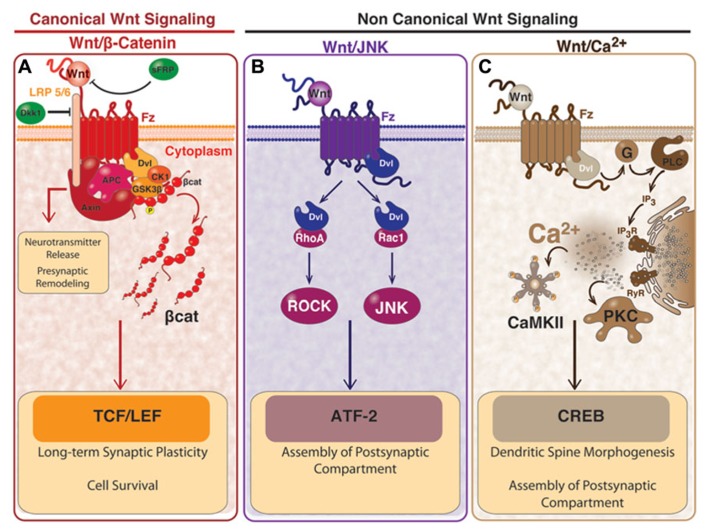
**Wnt signaling pathways and the effects in mature synapses. (A)** activation of canonical Wnt/β-catenin dependent pathway starts with the binding of Wnt ligand to Fz receptor and to the co-receptor LRP5/6, which induces the recruitment of Dvl and causes the inhibition of the “β-catenin destruction complex” formed by Axin, APC, GSK3β, and CK1. The inhibition of the “β-catenin destruction complex” causes the accumulation of β-catenin, which is no longer phosphorylated and it translocates to the nucleus where it activates transcription of Wnt target genes mediated by TCF/LEF. Canonical Wnt pathway participates in synaptic plasticity and cell survival. A divergent pathway involving proteins of the “β-catenin destruction complex” participates in the clustering of pre-synaptic proteins and the neurotransmitter release. The Wnt/β-catenin pathway can be blocked by Dkk-1 which binds to LRP, or through the sFRP which binds directly to Wnt ligand. **(B)** In the non-canonical Wnt/JNK pathway, activation of Dvl by the binding of Wnt to Fz, induces the activation of Rho and Rac small GTPases. Activation of Rho signals through ROCK and the activation of Rac signals through the JNK. This pathway is involved in the clustering of post-synaptic proteins and can also lead gene transcription mediated by ATF2. **(C)** In the non-canonical Wnt/Ca^2^^+^ pathway, the binding of Wnt to Fz, activates the heterotrimeric G-proteins which in turn signal through PLC and IP_3_ to induce the release of the intracellular Ca^2^^+^ and the activation of PKC and CaMKII. In this pathway, the transcription factor CREB is activated. Wnt/Ca^2^^+^ pathway is involved in the clustering of post-synaptic proteins and the dendritic spine morphogenesis. This figure is a courtesy of Felipe Serrano, Ph.D. student.

To date there is an enormous amount of information about Wnt signaling components and how they are compromised in different phenotypes (**Figure 1**). Historically, Wnt proteins has been classified as either canonical or non-canonical (Gordon and Nusse, 2006). For instance, Wnt1, Wnt3a, Wnt7a/b, and Wnt8 are common activators of the canonical pathway, while Wnt4, Wnt5a, and Wnt11 are mainly activators of the non-canonical pathway (Gordon and Nusse, 2006; MacDonald et al., 2009; Kikuchi et al., 2011). However, this traditional differentiation seems to be not as simple as was initially defined and more aspects should be considered. The fact that there are many different Wnt ligands and more than 15 different receptors and co-receptors allows enormous possibilities of interaction. Moreover, as result of these interactions, different intracellular cascades could be activated which makes that the cellular response turns difficult to be predicted (van Amerongen and Nusse, 2009). Furthermore, some evidences in the literature suggest that the activation of canonical or non-canonical pathway in a cell by a particular Wnt ligand may depend on the cellular context and the specificity by which Wnt binds to the receptor and co-receptor, and is not a property of the ligand itself (Mikels and Nusse, 2006b; Grumolato et al., 2010). It has been shown that Wnt ligands can also compete for the binding to specific receptors, causing the inhibition of the reciprocal signaling pathway (Grumolato et al., 2010). Despite this, some combinations of Wnt ligand-Fz receptor allow to predict the activation of a specific pathway. For example, the binding of Wnt3a ligand to Fz1 receptor activates the canonical pathway in PC12 cells (Chacon et al., 2008). Furthermore, if the Fz receptor binds to the co-receptor LRP5/6, it determines the activation of the canonical pathway, but if Fz binds to the co-receptor ROR1/2 the non-canonical pathway is activated instead.

### CANONICAL Wnt/β-CATENIN PATHWAY

The first Wnt signaling pathway identified was the “canonical pathway” (**Figure 1**). This pathway is also called Wnt/β-catenin pathway since the induction of some embryonic structures in *Xenopus* (McMahon and Moon, 1989a) and the transformation of mouse mammary cells (Wong et al., 1994) are strongly correlated with the increment in β-catenin/TCF levels (Shimizu et al., 1997). The activation of the Wnt/β-catenin pathway has been prominently involved in regulating cell differentiation and proliferation (Toledo et al., 2008; Niehrs, 2010; Wray and Hartmann, 2012) controlling growth and cell fate specification during development (Cadigan and Nusse, 1997). Since deregulation of β-catenin/TCF levels is a relevant feature of a variety of cancers (Reya and Clevers, 2005), this aspect of Wnt signaling is, to date, the most investigated (Kim et al., 2013).

In steady-state or in the absence of Wnt stimulation, the cytoplasmic cellular levels of β-catenin are low since casein kinase-1α (CK-1α) and glycogen synthase kinase-3β (GSK-3β) sequentially phosphorylates the protein, targeting β-catenin for ubiquitination and proteasome degradation (Aberle et al., 1997; Liu et al., 2002; Nusse and Varmus, 2012). Instead, in the presence of Wnt ligand, Wnt binds both Fz and LRP5/6 forming a Wnt-receptor complex that recruits the protein disheveled (Dvl), which oligomerizes in the plasma membrane making a platform for the recruitment and allocation of the “β-catenin destruction complex” (Bilic et al., 2007). This complex is formed by the scaffold protein Axin, GSK-3β, CK-1α, and the tumor suppressor adenomatous polyposis coli (APC; Cliffe et al., 2003; Schwarz-Romond et al., 2007; Gao and Chen, 2010). Once the complex is recruited, CK-1α phosphorylates LRP5/6 which causes the inhibition of the “β-catenin destruction complex” (**Figure 1**). As consequence of this inhibition, β-catenin is stabilized and accumulated in the cytoplasm and can enter to the nucleus to activate the transcription of Wnt target genes (Logan and Nusse, 2004) under the control of the TCF/LEF transcription factors (T-cell factor, TCF /lymphoid enhancer factor, LEF; Clevers and Nusse, 2012). There are several Wnt target genes that are activated in this process, including c-Myc, cyclin D1, Axin2, and Ca^2^^+^-calmodulin-dependent protein kinase type IV (CamKIV; Toledo et al., 2008; Arrazola et al., 2009; Hodar et al., 2010; Nusse and Varmus, 2012).

### β-CATENIN INDEPENDENT PATHWAY OR THE NON-CANONICAL PATHWAY

There are at least two other pathways activated by Wnt ligands which effects are independent of β-catenin. These pathways, known as the “non-canonical pathways,” are grouped according the Wnt receptor and co-receptors involved, and also by the intracellular signal triggered: the planar cell polarity (PCP) pathway and the Ca^2^^+^ pathway (**Figure 1**). The Wnt/PCP pathway was first identified in *Drosophila* where it regulates tissue polarity and cell migration during development (Adler, 2002; Veeman et al., 2003). The Wnt/PCP pathway signals through c-Jun N-terminal kinase (JNK) to control cell polarity, hence it is also known as the Wnt/JNK pathway (Boutros et al., 1998; Yamanaka et al., 2002). In this pathway, the binding of Wnt to the Fz receptor on the membrane surface is followed by the activation of Rho and Rac small GTPases which in turn stimulate ROCK and JNK, respectively (**Figure 1**). The downstream effect of this pathway is the regulation of the cytoskeletal organization, cell motility, and gene expression by JNK-dependent transcription factors, for example the activating transcription factor 2 (ATF2; **Figure 1**), with concomitant activation of its target genes (Simons and Mlodzik, 2008; Kikuchi et al., 2011).

The Wnt/Ca^2^^+^ pathway is mostly a G-protein dependent signaling pathway (Slusarski et al., 1997a; Kohn and Moon, 2005). The activation of the Wnt/Ca^2^^+^ pathway requires the binding of Wnt to Fz on the membrane surface to stimulate heterotrimeric G-proteins (Slusarski et al., 1997a,b), which activates, in turn, the phospholipase-C (PLC). As a result, PLC in turn stimulates the generation of diacylglycerol (DG) and inositol-1,4,5-thriphosphate [Ins(1,4,5)P_3_]. This last one triggers the increase of the intracellular Ca^2^^+^-release, decreases cyclic guanosine monophosphate (cGMP), and activates the Ca^2^^+^-calmodulin-dependent protein kinase II (CaMKII), calcineurin, and protein kinase-C (PKC; Veeman et al., 2003; Kohn and Moon, 2005; Montcouquiol et al., 2006). In this process several transcription factors are activated, including the cAMP Response Element-Binding Protein-1 (CREB; **Figure 1**).

The classification of individual Wnt proteins in canonical and non-canonical is used now to indicate the activation of a signaling pathway, either β-catenin dependent or independent (Mikels and Nusse, 2006a, b; van Amerongen and Nusse, 2009). It has been shown that the induction of canonical or non-canonical pathway depends on the particular co-receptor activated (Grumolato et al., 2010). Moreover, the activation of a specific co-receptor by a Wnt ligand involves a common mechanism that requires the recruitment of Dvl, Axin, and GSK-3β in order to trigger either canonical or non-canonical pathways (Grumolato et al., 2010). Strikingly, Wnt ligands can compete for the Fz receptor at the cell surface and cause inhibition of the reciprocal pathway (Grumolato et al., 2010), therefore providing another control point to determine the specificity of signaling.

## Wnt EXPRESSION IN THE POSTNATAL CNS

During the establishment of neuronal connectivity at early and late postnatal development, molecules such as the brain-derived neurotrophic factor (BDNF), bone morphogenetic protein (BMP), and Wnts act in the postnatal nervous system to help the recently formed synapses to establish and consolidate the new cellular interactions, and later to lead synaptic remodeling and plasticity throughout life (Lu, 2003; Marques, 2005; Speese and Budnik, 2007). BDNF is not only required during early development for neuronal survival and function (Duman and Voleti, 2012), but also in the adult brain to improve synaptic transmission, long-term potentiation (LTP), and ocular dominance plasticity (Magby et al., 2006; Maya Vetencourt et al., 2008; Duman and Voleti, 2012). Recently, it has been demonstrated that BDNF is a direct target of the canonical Wnt pathway in glial cells (Yi et al., 2012). Moreover, an activator of Wnt induces BDNF in retina glial cells and Wnt3a induces BDNF in a retina ganglion cell line (Seitz et al., 2010; Fragoso et al., 2011), linking both BDNF and Wnt signaling pathways in common effectors actions (Yi et al., 2012). Like BDNF, Wnt proteins remain expressed in the postnatal brain where they play a fundamental role modulating the connectivity between pre- and post-synaptic regions, participating in the remodeling of synaptic structures and regulating synaptic function (Inestrosa and Arenas, 2010; Wisniewska, 2013). Next we briefly discuss the histological evidence that shows the expression of the Wnt pathway in the postnatal brain (for more detail review (Oliva et al., 2013)).

The Wnt ligands and several components of the Wnt pathway have been identified in the adult brain in the major subdivisions of the cerebral cortex, in the olfactory bulb (OB), and olfactory related areas, in the hippocampal formation, neocortex, and in the thalamus (Shimogori et al., 2004; Wayman et al., 2006; Cerpa et al., 2008; Davis et al., 2008; Gogolla et al., 2009). *In situ *hybridization shows that expression of different Wnts is particularly high in those areas where the neurons are continuously renewed, such as OB and DG (Shimogori et al., 2004) and during the period of dendrite development in the hippocampus (Wayman et al., 2006).

The presence of some components of the Wnt signaling in the thalamus during postnatal life is also relevant (Shimogori et al., 2004; Wisniewska, 2013), since the thalamus is the relay for most of the sensory information to the cortex. A recent work shows that β-catenin regulates the expression of a group of genes in the thalamus (Wisniewska et al., 2012). These highly expressed genes encode for proteins involved in neuronal excitability, including voltage-gated ion channels, neurotransmitter receptors, synaptic vesicle proteins, and synaptic structural proteins. As such, they underlie cell membrane conductances of Ca^2^^+^, K^+^, and Cl^-^ ions (in the case of the GABA receptor) and directly propagate, inhibit, or modify electric signals. Hence, β-catenin could be relevant to determine the degree of excitability in the thalamic neurons by regulating the expression of those genes and influencing the thalamocortical circuit activity (Wisniewska et al., 2012; Wisniewska, 2013). Since sensory information is continuously processed during the lifespan of an animal it is likely that Wnt signaling plays an important role in thalamocortical connectivity based on experience. However, further studies are required to address this important issue.

Whether Wnt ligands expression is crucial to determine the final structural and functional pattern in a mature circuit of the adult brain is matter of speculation. However, the presence of Wnt components in many brain areas, including those that are actively participating in neurogenesis, or those that are implicated in sensory processing and superior cognitive processes, suggest that Wnt pathway is involved in more than only structural functions. In the next sections we review the evidences that relate Wnt to active structural and functional processing in mature synapses, and we discuss the importance that this can have to modulate active depending processes, such as synaptic plasticity.

## Wnt FUNCTION IN THE DEVELOPING AND MATURE CNS

### ROLE OF Wnt SIGNALING IN THE PRE-SYNAPTIC TERMINAL

Wnt ligands have been linked to the assembly of structural components in the pre-synaptic compartments. For example, Wnt7a can act as a retrograde signal from granular cells (GC) in the cerebellum to induce pre-synaptic differentiation in the mossy fiber (MF), working as a synaptogenic factor (Hall et al., 2000; Ahmad-Annuar et al., 2006). Wnt7a induces axonal spreading, increases the size of the growth cone and its branching (Lucas and Salinas, 1997; Hall et al., 2000) and probably contributes to the formation of the active zones because it increases the clustering of synapsin I, a synaptic vesicle component (Hall et al., 2000). Wnt7a also induce clustering of synaptophysin, synaptotagmin, and SV-2 without affecting the clustering of post-synaptic proteins like the post-synaptic density protein-95 (PSD-95) (Cerpa et al., 2008). Moreover, a mutant mouse deficient in Wnt7a shows a delayed synaptic maturation (Hall et al., 2000) supporting the notion of the ligand Wnt7a modulating the pre-synaptic compartment. On the other hand, lithium, a pharmacological inhibitor of GSK-3β, mimics the effect of Wnt7a on axonal spreading and branching in cerebellar granular cells *in vitro*, suggesting that GSK-3β also participates in synaptic maturation (Lucas and Salinas, 1997). However, GSK-3β is not required for the clustering of pre-synaptic vesicle proteins induced by Wnt7a (Cerpa et al., 2008). Instead, clustering induction correlates with β-catenin stabilization, does not involve gene expression, and is also mimicked by Wnt3a. It has been suggested that Wnt7a requires Dvl1 to regulate the normal recycling rate of synaptic vesicles, and the deficiency of both proteins (Wnt7a/Dvl1 double null mutant mice) significantly reduces the miniature excitatory post-synaptic current (mEPSC) frequency, an indication of a defect in the neurotransmitter release (Hall et al., 2000; Ahmad-Annuar et al., 2006). Additionally, Wnt7a increases the frequency of mEPSC and decreases the rate of paired-pulse facilitation (PPF) (Cerpa et al., 2008), a protocol used to distinguish the involvement of the pre-synaptic from the post-synaptic terminal (Foster and McNaughton, 1991). Also, using FM dye it has been proved that Wnt7a stimulates the recycling and accelerates the exocytosis of synaptic vesicles (Hall et al., 2000; Ahmad-Annuar et al., 2006; Cerpa et al., 2008). Wnt7b and Wnt3a also increase the number of pre-synaptic puncta suggesting a role for these ligands in the pre-synaptic assembly (Ahmad-Annuar et al., 2006; Cerpa et al., 2008; Davis et al., 2008). Altogether these evidences support the role of Wnt in the maturation and function of pre-synaptic terminals.

Wnt7a has been also involved in receptors trafficking since it increases the number and size of co-clusters of the pre-synaptic α_7_-nicotinic acetylcholine receptors (α_7_-nAChR) and APC in hippocampal neurons, as well as it modulates the α_7_-nAChR trafficking to the nerve terminal (Farias et al., 2007). This evidence indicates that Wnt pathway is actively involved in the receptors functional availability in the pre-synaptic terminal.

Most of the ligands that are able to modulate pre-synaptic differentiation have shown to activate the Wnt/β-catenin signaling pathway. Despite that, nanomolar concentrations of Wnt3a which also modulates the recycling and exocytosis of synaptic vesicles in the hippocampal synapses, increases the frequency of mEPSC through a mechanism that involves Ca^2^^+^ entrance (Cerpa et al., 2008; Avila et al., 2010), suggesting a cross-talk between canonical Wnt pathway and the Wnt/Ca^2^^+^ signaling (Avila et al., 2010). This evidence implies that some of the components associated with the non-canonical pathway may also be involved in the functionality of the pre-synaptic nerve terminal. Altogether these evidences suggest that Wnt ligands that activate canonical Wnt pathway are involved in the pre-synaptic structure assembly and participate during consolidation of synaptic connectivity.

### ROLE OF Wnt SIGNALING IN THE POST-SYNAPTIC REGION

Wnt signaling can actively modulate the structural and functional assembly of the post-synaptic terminal. For instance, Wnt5a modulates excitatory synaptic transmission (Cerpa et al., 2010, 2011), specifically up-regulating excitatory synaptic currents through NMDA receptors (NMDARs), but not AMPA receptors (AMPARs), through activation of PKC and JNK, and facilitating the induction of excitatory LTP (Cerpa et al., 2011). Moreover, Wnt5a actively modulates inhibitory synapses in the hippocampus, increasing the turnover of GABA_A_ receptor (GABA_A_Rs) in the surface and increasing GABA currents by a post-synaptic mechanisms that involved Wnt/Ca^2^^+^/CaMKII (Cuitino et al., 2010). Wnt5a also participates in the dendritic spine morphogenesis, inducing *de novo* dendritic spines and causing an increase in the volume and density of pre-existent spines, enhancing the efficacy of hippocampal glutamatergic synapses (Varela-Nallar et al., 2010). Wnt5a also induces the increment of calcium in the synaptic puncta of cultured hippocampal neurons, suggesting the activation of Wnt/Ca^2^^+^ signaling pathway (Varela-Nallar et al., 2010) through a mechanism that involved fast phosphorylation of CaMKII (Farias et al., 2009). Wnt5a also modulates post-synaptic protein assembly by increasing the clustering of PSD-95, without affecting the total levels of this protein, via activation of Wnt/JNK signaling pathway (Farias et al., 2009).

Among the non-typical functions attributable to the ligands, we consider some examples. It has been shown that Wnt3a induces clustering of AChRs in motoneurons during the neuromuscular innervation (Henriquez et al., 2008). This effect requires Dvl1 and agrin, a protoglycan released by motoneurons, but does not involve Wnt/β-catenin pathway. Instead, Wnt3a induces AChRs aggregation through activation of Rac1 (Henriquez et al., 2008). However, Wnt3a through the activation of Wnt/β-catenin pathway inhibits agrin-induced AChR clusters, suggesting that Wnt signaling dynamically regulates the post-synaptic assembly during the establishment of the neuromuscular junction (Wang et al., 2008).

In another example, Wnt7a has also been shown to regulate the post-synaptic compartment by stimulating the morphogenesis and function of excitatory dendritic spines, without affecting the inhibitory connectivity in the hippocampus (Ciani et al., 2011). It has been shown that Wnt7a requires Dvl expression to induce spine growth in pre-existed post-synaptic spines, as well as the activation of CaMKII to induce spine maturation (Ciani et al., 2011). On the other hand, it has been shown that the ligand Wnt7b and Dvl, independently of Wnt/GSK-3β, can activate Rac and downstream JNK to increase dendritic length and branching in immature hippocampal neurons (Rosso et al., 2005).

Interestingly, while the activation of canonical pathway by Wnt3a, Wnt7a, or Wnt7b induces synaptogenesis through β-catenin stability, activation of non-canonical pathway through Wnt5a negatively regulates synaptogenesis (Davis et al., 2008), which could constitute a competitive mechanism to balance the synapse formation in the brain. Altogether these evidences support the idea of a new role for traditional canonical Wnt ligands, which associated with the non-canonical pathway may also be involved in the functionality of the post-synaptic region.

## Wnt IN NEURONAL PROCESSING

In the previous sections we mainly reviewed the role of Wnt signaling pathways in synaptic structure and in particular functional changes through development. In this section we address how Wnt signaling may be relevant for physiological functions during activity and non-activity dependent processes.

### Wnt SIGNALING IN BASAL NEURONAL ACTIVITIES

Several evidences in the literature show that Wnt signaling is constitutively released during basal synaptic activities. Using sFRP1, a physiological Wnt signaling inhibitor, and using a reporter cell line that expresses GFP, it has been shown that hippocampal cultures are spontaneously secreting measurable amounts of endogenous Wnt (Rosso et al., 2005). Wnt is also constitutively released in slices and specifically regulates the function of NMDAR (Cerpa et al., 2011). Moreover, it has been shown that using PKC or JNK inhibitors to block the downstream signaling, the basal NMDAR synaptic neurotransmission is reduced, suggesting that Wnt is using non-canonical components of Wnt pathway to maintain glutamatergic neurotransmission (Cerpa et al., 2011). sFRP1 also reduces the amplitude of evoked fEPSP in hippocampal slices, confirming that Wnt is being released to maintain the basal tone of synaptic activity (Varela-Nallar et al., 2010). These evidences strongly support the notion that during basal activity Wnt molecules are being spontaneously released to keep functional connectivity. Indeed, it has been observed that local inhibition of mossy fibers with sFRP decreases the levels of Wnt7a/b protein, suppressing the increased effect that an enriched ambient has on the synapses number (Gogolla et al., 2009). Interestingly, in animals housed in control conditions, sFRP also reduces the synapses number, suggesting that Wnt signaling is required to generate structural plasticity (Gogolla et al., 2009). Recently, we identify a very dynamic effect of Wnt ligands, modulating the spike rate in brain slices *in vitro*. Using a slice model in mice that can generate spontaneous oscillations we recently demonstrated that Wnt3a perfused into the recording solution is able to modulate the spike frequency, while antibodies against Wnt ligands significantly affect the oscillation (Oliva et al., 2013). The possibility that Wnt is being constantly released to exert functional effects on the cells in a network is intriguing, as well as exciting possibility. Nevertheless, the function of the released Wnts in neuronal network remains to be resolved.

Several studies have shown that the neocortex and the hippocampus are electrically active even from embryonic stages, and this early neuronal activity has a potent trophic role, mediating neuronal survival (Golbs et al., 2011) and maturation of molecular, cellular, and structural features of cortical circuits (Owens and Kriegstein, 2002). This spontaneous activity in the form of spontaneous action potentials plays a fundamental role in the fine tuning of connectivity in several regions of the brain (Masland, 1977; Ben-Ari et al., 1989; Yuste et al., 1992; Feller et al., 1996; Garaschuk et al., 1998; Komuro and Rakic, 1998; Moody and Bosma, 2005; Mire et al., 2012). The establishment of early network patterns during the first weeks of life has different functions and the underlying mechanisms are different of those of mature network. The presence of molecules such as Wnt and BDNF during particular early developmental moments may help in the establishment and consolidation of the recently formed synapses (Lu, 2003; Marques, 2005; Speese and Budnik, 2007), contributing to the circuitry maturation and consolidating the rhythmic activity that can sustain adult behavior (Egorov and Draguhn, 2012). How Wnt signaling is contributing to the establishment of mature neural circuits, is still matter of speculation, but based on the histological and functional evidences, it is expected to be important.

### Wnt SIGNALING IN ACTIVITY-DEPENDENT PROCESSESS

Wnt ligands and receptors are expressed in areas of the brain that undergo plasticity, suggesting that during an activity-dependent process Wnt is released to regulate synaptic transmission and ulterior structural modifications. Dendritic spines, direct targets of the Wnt action, have been considered as one of the first visible effectors of plasticity (experience dependent)-induced changes (Alvarez and Sabatini, 2007; Holtmaat and Svoboda, 2009; Bosch and Hayashi, 2012). During development, synaptogenesis contributes to establish the connectivity within neuronal circuits (Holtmaat and Svoboda, 2009). Later, during postnatal development and adolescence, activity-dependent spine maintenance or elimination contributes to the remodeling of neuronal circuits (Alvarez and Sabatini, 2007). In this section we review the evidence of Wnt signaling as one of the signaling pathways responsible for structural dendritic changes during activity-dependent plasticity.

Using high K^+^ solution, a solution used to mimic the depolarization caused by persistent neuronal activity (Ryan et al., 1993), Yu and Malenka found that the increment in the number and length of dendritic branches in culture hippocampal neurons depends on β-catenin availability (Yu and Malenka, 2003) and showed that this effect is reduced by Dkk-1. Using a GFP-reporter cell line to detect Wnt they demonstrated that neuronal depolarization with K^+^, increases Wnt secretion from hippocampal neurons but not from astrocytes, contributing to the idea that changes in dendritic arborization depend on activity-induced Wnt release (Yu and Malenka, 2003). Moreover, CaMKIV that has been also shown to regulate depolarization-dependent dendritic growth requires β-catenin (Redmond et al., 2002; Yu and Malenka, 2003).

Other evidences show that Wnt released in an activity-dependent manner modifies the structure and function of dendritic branches. Neuronal activity induced by high K^+^ sequentially activates NMDARs, CaMKK, CaMKI, Ras, MEK/ERK, and CREB-dependent transcription of Wnt2 (Wayman et al., 2006), indicating that Wnt2 could be a CREB-responsive gene. Indeed, Wnt2 can stimulate dendrite development by itself (Wayman et al., 2006). Along these lines, another report showed that the Wnt inhibitor WIF, which prevents that Wnt binds its receptor Fz (Han and Lin, 2005), suppresses dendritic arborization induced by high K^+^. Based on a previous report indicating that CaMKIV initiates the cascade culminating in CREB activation and dendritic maturation (Redmond et al., 2002), it is plausible that this effect is mediated through CREB-dependent Wnt (Wayman et al., 2006). This evidence strongly suggests that sustained activity can induce synthesis of more Wnt to potentiate the cellular response. Altogether, these evidences suggest an active role of Wnt in determine the strength and remodeling of the synapse in response to an activity-dependent process.

Using a more physiological paradigm to mimic neuronal depolarization, such as theta burst stimulation or high frequency stimulation (HFS) to induce LTP (Larson et al., 1986), it has been also shown an increases in the expression of Wnt signaling components and the Wnt release in an activity-dependent manner. LTP induces changes in the mRNA levels and the immunostaining pattern of several Wnt proteins, such as Wnt3a, Fz4, β-catenin, and Dvl3 (Chen et al., 2006). LTP also induces accumulation of β-catenin in the nucleus and the increment of several Wnt-target genes, confirming the activation of Wnt/β-catenin/TCF pathway by tetanic stimulation (Chen et al., 2006). In fact, the size and number of Wnt3a puncta diminishes after tetanic stimulation, suggesting that Wnt3a is being released in a process that depends of NMDAR activation (Chen et al., 2006). Moreover, the treatment with a specific antibody against Wnt3a shows a significant reduction on LTP, while incubations with lithium enhances LTP, also showing that Wnt3a is endogenously released to activate Wnt/β-catenin pathway (Chen et al., 2006). These studies strongly suggest that the presence of Wnt ligand and/or the activation of the canonical pathway are required to induce LTP.

On the other hand, a recent report has shown that neuronal activity decreases the expression of sFRP3, releasing the Wnt pathway from its natural inhibitor (Jang et al., 2013). The reduction in sFRP3 is essential for the activity-dependent neuronal maturation process in the hippocampus, increasing neurogenesis (Jang et al., 2013), a mechanism that can be used to overcome neuronal death. This evidence suggests an important effect of neural activity on the neurogenesis induction, process that appears to be under tight control of Wnt signaling. More studies are required to confirm this hypothesis.

### STUDYING THE FUNCTION OF Wnt SIGNALING *IN VIVO*

The evidences discussed here reveal that sustained depolarization activates Wnt signaling, increases synaptic transmission and facilitates LTP in hippocampal brain slices and in cultured neurons. Wnts also have a significant effect controlling the basal activity on brain circuits, suggesting an important role for Wnt signaling in the regulation of spontaneous and activity-dependent plasticity (Inestrosa and Arenas, 2010; Budnik and Salinas, 2011). However, there is still a lack of *in vivo* evidence about how Wnt signaling may affect physiological synaptic processes, for example during active behavior.

Recent studies have suggested a role for the Wnt/β-catenin pathway in the memory formation of adults (Maguschak and Ressler, 2008, 2011), while, deregulation of the Wnt/β-catenin pathway has been implicated in the Alzheimer’s diseases (ADs) pathogenesis (Moon et al., 2004), which is associated with memory loss. However, it remains unknown how Wnt signaling is involved in learning and memory processes in the adult. Recently, it has been reported that stereotaxic injection of the Wnt antagonist Dkk-1 in the basolateral amygdala interferes with the long-term memory consolidation without affecting short-term memory (Maguschak and Ressler, 2011). Remarkably, administration of Wnt1 during fear memory formation also interferes with the long-term memory consolidation in the amygdala (Maguschak and Ressler, 2011). The Wnt1 administration avoids the transient decrease in the Wnt1 mRNA that occurs immediately after fear conditioning, suggesting that the rapid decrease in the expression of Wnt1 might be critical for the fear memory formation (Maguschak and Ressler, 2011). However, not only the Wnt1 mRNA but also several genes related to Wnt signaling are rapidly downregulated during fear learning and normalized during memory consolidation (Maguschak and Ressler, 2011). Also, during fear memory consolidation in the amygdala there is a transient increment of β-catenin mRNA levels, while the β-catenin deletion specifically affects fear memory consolidation of adult mice (Maguschak and Ressler, 2008).

On the other hand, a selective increment in the hippocampal levels of Wnt7 and Wnt5a, but not Wnt3 isoform, during consolidation and recall of spatial memory has been reported (Tabatadze et al., 2012). The levels of Wnt7 in the hippocampus of animals trained in a water maze hidden platform, are enhanced in comparison to the animals trained with a visible platform (Tabatadze et al., 2012). Despite in the visible platform the memory acquisition is better, animals trained in the hidden platform have better retention memory after 30 days of training, that correlates with elevated levels of Wnt7 (Tabatadze et al., 2012). The cells that concentrate the expression of Wnt7 are the granular cells in the DG, showing a correlation between the expression of Wnt7 in this region with the establishment of the long-lasting memory (Tabatadze et al., 2012).

Recently, it has been reported that the activation of Wnt/β-catenin pathway is also required for memory consolidation of a hippocampal-dependent object recognition task (Fortress et al., 2013). Intrahippocampal administration of Dkk-1 avoids familiar object recognition and generates a strong reduction in Wnt-related proteins such as β-catenin, Cyclin D1, c-myc, and Wnt7, evidencing the role of canonical Wnt signaling in hippocampal consolidation memory (Fortress et al., 2013). In another study it has been shown that an aged APC heterozygous knockout (APC^+^^/^^-^) mice exhibit a severe insufficiency in the working memory performance suggesting that a correct regulation of Wnt signaling is determinant for the appropriate functioning of the CNS (Koshimizu et al., 2011). More evidence has recently involved Wnt signaling in olfactory memory in *Drosophila*, showing that *armadillo* (homolog to β-catenin), *wingless* (Wnt ligand), and *arrow* (co-receptor) are necessary for long term memory formation (Tan et al., 2013), indicating that these mechanisms are evolutionary conserved among spacies.

Altogether, these evidences support the idea that Wnt/β-catenin pathway is involved in long-term memory consolidation and that the activation of Wnt signaling in response to a sensory stimulus induces functional long-lasting changes improving the animal’s performance. In the next section, we review the evidences in the literature showing that deregulation of the Wnt pathway might be related to major neurological disorders.

## Wnt SIGNALING DYSFUNCTION IN NEURODEGENERATIVE AND MENTAL DISORDERS

**FIGURE 2:**
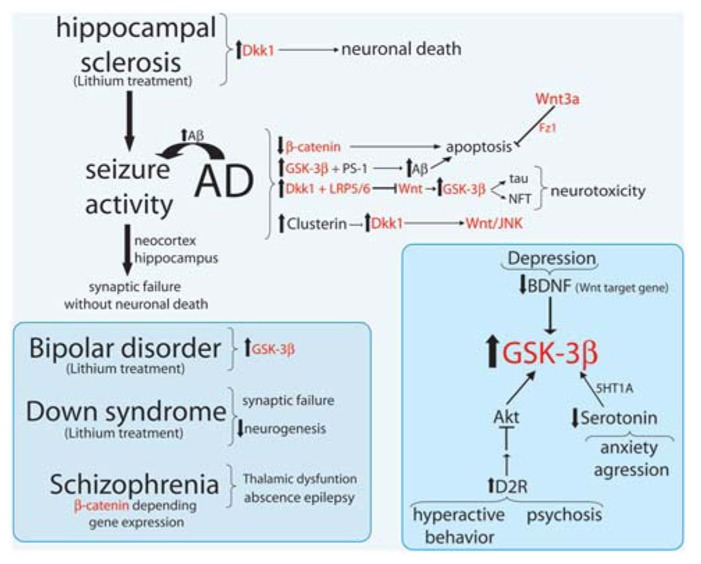
**Major neurological disorders and their relationship with Wnt signaling components.** Summary scheme of Wnt signaling components (in red) involved in Alzheimer disease (AD), seizure activity (epilepsy), hippocampal sclerosis, bipolar disorder, down syndrome, and Schizophrenia.

During the last decades, increasing data has suggested that the progression of a neurological disease is the result of severe neuroanatomical abnormalities. However, more evidences shows that several changes in the synaptic connectivity such as the imbalance between excitation/inhibition may result into a cascade of multiple effects at the neural network level which in many cases precedes cell degeneration or death (Hickey et al., 2008; Hermann et al., 2009; Palop and Mucke, 2010; Penzes et al., 2013). Excitatory/inhibitory balance critically regulates cortical network function, neural network activity, and behaviors associated with psychiatric disorders (Penzes et al., 2013). Multiple lines of evidence implicate excitatory and inhibitory synaptic circuits in the cortex, striatum, and hippocampus as key cellular substrates for pathogenesis in these disorders. In fact, major neurological diseases such as autism, schizophrenia (SZ), bipolar disorder, and major neurodegenerative disorders including AD, Huntington’s disease (HD), and Parkinson’s disease (PD) share these characteristics, that the neurodegenerative diseases show progressive neuronal loss. In this section we review the most recent studies related to the progression of the most common neurological diseases in which Wnt signaling pathway and its components has been suggested to be relevant (**Figure 2**).

### ALZHEIMER’S DISEASE AND Wnt SIGNALING

Alzheimer’s disease is a neurodegenerative disorder characterized by progressive deterioration of the individual cognitive functions, mainly caused by synaptic damage and neuronal death in specific regions of the brain (Selkoe, 2001; Mattson, 2004; Walsh and Selkoe, 2004). Distinctive features of the AD brains are the presence of senile plaques, composed by extracellular deposits of amyloid-β (Aβ) peptides and the neurofibrillary tangles (NFT), composed by intracellular aggregates of hyper-phosphorylated tau protein (Mayeux and Stern, 2012). Despite there is not clarity yet about the triggering factors for AD, there are some candidates which are also common factors to several other neuronal diseases (Jope and Johnson, 2004), among which are components of the Wnt signaling machinery (**Figure 2**). One of them is the GSK-3β (Hooper et al., 2008), a constitutively active protein and whose expression is increased in the hippocampus of AD patients (Blalock et al., 2004). Over-expression of GSK-3β in mice prevents the induction of LTP and reduces the spatial learning (Hernandez et al., 2002; Hooper et al., 2007), linking the characteristic memory failure in AD to the increment in GSK-3β. The same transgenic mouse exhibits tau hyperphosphorylation, low β-catenin in the nucleus, and neurodegeneration by NFT (Lucas et al., 2001), features that can be prevented by chronic lithium exposure (Engel et al., 2006a, b). In fact, GSK-3 activity mediates Aβ production from its precursor amyloid precursor protein (APP; Phiel et al., 2003; Ryder et al., 2003). It has been shown that GSK-3β binds presenilin-1 (PS-1), a protein involved in the proteolytic cleavage of the APP, to produce the Aβ peptide. The expression of a mutant PS-1 shows an increment in GSK-3 activity and in the hyperphosphorylated tau protein in cells (Takashima et al., 1998; Zhang et al., 1998). In cultured neurons the toxicity mediated by Aβ depends on increased GSK-3β activity, but it reverses when GSK-3β expression or its activity is blocked (Takashima et al., 1993, 1996; Busciglio et al., 1995; Alvarez et al., 1999).

We previously demonstrated that activation of Wnt signaling inhibits GSK-3β, and leads to neuroprotection in both hippocampal cultured neurons and *in vivo* transgenic model of AD (Garrido et al., 2002; Inestrosa et al., 2002; De Ferrari et al., 2003; Alvarez et al., 2004; Quintanilla et al., 2005). A marked decrease in the levels of β-catenin has been found in the brain of early onset familial AD patients with PS-1 mutations (Zhang et al., 1998). It has been shown that PS-1 form a complex with β-catenin, similarly to how β-catenin is associated to GSK-3 and to APC (Zhang et al., 1998). The inhibition of the β-catenin/TCF pathway enhances the vulnerability to apoptosis (Zhang et al., 1998), suggesting that when β-catenin is stabilized and TCF is activated, it could lead to neuronal survival. Moreover, Li and colleagues found that tau hyperphosphorylation, which inhibits competitively phosphorylation of β-catenin by GSK-3β, protects the cells from apoptosis (Li et al., 2007). Wnt3a can also protect from Aβ-induced apoptosis through binding Fz1 but no Fz2 (Chacon et al., 2008) and the genetic variations in LRP-6 have been associated with late-onset AD (De Ferrari et al., 2007), supporting the evidences that alterations in Wnt/β-catenin signaling could be involved in AD.

Another molecule of the Wnt pathway involved in the AD phenotype is Dkk-1, that is the cross-link between the pathway to survival/apoptosis that relate Aβ and Wnt signaling (**Figure 2**). While canonical Wnt/β-catenin endorses cell survival, Aβ induces the expression of the natural antagonist Dkk-1. In AD brains there are an up-regulated expression of Dkk-1 compared to healthy individuals (Caricasole et al., 2004), while the chronic overexpression of Dkk-1 in transgenic mice causes an age-related tau phosphorylation and cognitive deficits (Killick et al., 2012). During Aβ exposure there is an induction of Dkk-1 expression that depends on p53 (Wang et al., 2000; Zhang et al., 2002; Killick et al., 2012). Since Dkk-1 binds LRP5/6 to inhibit Wnt signaling, the lack of inhibition of GSK-3β facilitates tau hyperphosphorylation and the NFT formations, leading neurotoxicity and apoptosis caused by Aβ peptides (Caricasole et al., 2004). Dkk-1 knock-down almost completely avoid neuronal death and tau phosphorylation, blocking neurotoxicity induced by Aβ (Caricasole et al., 2004). Dkk-1 can also reversibly reduce the amount of synaptic proteins and the number of active pre-synaptic sites, by inducing synaptic disassembly of both pre- and post-synaptic sites (Purro et al., 2012). However, using an antibody anti-Dkk-1 the synaptic loss induced by Aβ can be blocked (Purro et al., 2012). Recently it has been reported that Dkk-1 expression increases in an age-dependent manner and causes the reduction of hippocampal neurogenesis. In neural progenitors with inducible loss of Dkk1 not only enhance neurogenesis and the TCF/LEF activity, but reverses the cognitive decline related to deleterious acquisition and memory consolidation (Seib et al., 2013). These evidences might have several implications for future treatments of dementias and neurodegenerative diseases, yet more studies are required.

New evidences also support a link between Aβ toxicity and Wnt pathway. Besides *APOE locus*, classically associated with susceptibility for late-onset AD, it has been identified a new susceptibility factor in late-onset AD, a loci at* CLU* (Lambert et al., 2009). Both APOE and CLU, which are considered the most abundant apolipoproteins in the CNS (Roheim et al., 1979), have been involved in Aβ clearance from the brain through the blood-brain barrier (DeMattos et al., 2004; Lambert et al., 2009). Clusterin, a product of CLU promotes cell survival, is increased in AD models (Killick et al., 2012) and is regulated by Wnt signaling via TCF1 (Schepeler et al., 2007). Suppression of the Wnt signaling pathway in colon carcinoma cells lead to up-regulation of CLU (Schepeler et al., 2007). The knock-down of clusterin protects against Aβ neurotoxicity and prevents the induction of Dkk-1 by Aβ (Killick et al., 2012). In contrast, Aβ targets clusterin allowing its intracellular accumulation and initiating the neurotoxicity process (Killick et al., 2012).

The C-terminal of Dkk-1 protein, which is the domain that antagonizes the canonical Wnt pathway through binding LRP5/6, is responsible for the activation of gene transcription of genes involved in AD-like pathology (Killick et al., 2012). Interestingly, Dkk-1 blocks Wnt/β-catenin signaling and activates the non-canonical Wnt/JNK pathway, leading the increment in c-Jun activity (Killick et al., 2012). Thus, activation of gene transcription in the presence of Aβ occurs due to that Dkk-1 activates Wnt/JNK pathway, which leads to tau phosphorylation. Moreover, clusterin and several genes from the Wnt/JNK signaling have been found in AD human-brain (Killick et al., 2012), supporting the idea that Aβ induces clusterin/Dkk-1/JNK pathway to produces neurotoxicity.

On the other hand, lithium an activator of Wnt/β-catenin signaling (Klein and Melton, 1996; Gould et al., 2004) indirectly induces protection against Aβ neurotoxicity in mice models of AD (Rockenstein et al., 2007). In a study from our laboratory we reported that *in vivo* activation of Wnt pathway by lithium reduces memory loss and Aβ aggregates in APP/PS1 double transgenic mice, an AD animal model (Toledo and Inestrosa, 2010). Lithium also reduces the level of tau aggregates and axonal degeneration in transgenic mice over-expressing human tau, suggesting that Wnt pathway can also control tautopathy progression (Noble et al., 2005). Moreover, lithium improves spatial memory after being decreased by Aβ fibrils injections (De Ferrari et al., 2003; Toledo and Inestrosa, 2010) and interferes with the cleavage of APP causing a reduction in Aβ levels (Phiel et al., 2003).

Interestingly, we observed that *in vivo* activation of Wnt signaling also improves episodic memory of APP/PS1 mice (personal observations). These results agree with recent findings showing that the activation of Wnt/β-catenin signaling through the inhibition of GSK-3β, can reverses the hippocampus-dependent learning deficits in a mouse model of fragile-X syndrome (Guo et al., 2012). More studies are required, at present, to determine the nature of this effect.

### Wnt SIGNALING IN EPILEPSY AND SEIZURES

It has been described that transgenic mouse with familial mutation associated to AD presents high levels of altered neuronal activity, indicated by the electroencephalographic studies and for the susceptibility to develop seizure activity in several brain regions, including neocortex and hippocampus (Palop et al., 2007). The same incidence has been found in AD patients (Menendez, 2005). This epileptiform activity *in vivo* occurs in absence of neuronal loss, supporting the hypothesis that an aberrant neuronal activity is a primary effect of the high Aβ levels and not a consequence of the neurodegeneration (Palop et al., 2007; Palop and Mucke, 2009). In a recent work it has been shown that the overexpression of the APP intracellular domain (AICD) increases the susceptibility to have spontaneous seizures, deficits in LTP and seizure-induced drugs generation (Vogt et al., 2011). Moreover, in these models the seizure susceptibility and severity depends on the levels of AICD; at higher AICD levels, most severe seizures (Vogt et al., 2011). These effects are independent of the levels of Aβ, because a mice overexpressing hAPP but with a mutation in AICD does not generates deficits in LTP or memory, despite the levels of Aβ (Galvan et al., 2006; Saganich et al., 2006). Both data are not contradictory and suggest that not only high levels of Aβ but also overexpressed AICD are required to cause seizure susceptibility. Interestingly, the overexpression of AICD increases the levels of active GSK-3β (Ryan and Pimplikar, 2005), something that also occur in AD patients (Hooper et al., 2008). Whether this evidence relates Wnt signaling with seizure susceptibility has not been demonstrated yet. However, cells with NFT inclusions and positives to active GSK-3β are allocated in brain regions where Wnt components have been well described (Pei et al., 1999).

On the other hand, an early expression of Dkk-1 has been found in patients with hippocampal sclerosis, characterized by temporal lobe epilepsy, severe neuronal loss and gliosis (Busceti et al., 2007). Like in hypoxia/ischemia *in vivo* and in excitotoxicity-related neuronal death *in vitro* (Cappuccio et al., 2005), the expression of Dkk-1 in hippocampal sclerosis correlates with the neuronal death (Busceti et al., 2007). In fact, the increase in Dkk-1 precedes neuronal death in both the olfactory neurons as in the hippocampus of rats that developed seizures induced by kainate (Busceti et al., 2007). Animals previously injected with lithium are protected against neuronal loss, but not against the kainate-induced seizures (Busceti et al., 2007). Intracerebral administration of Dkk-1 antisense almost completely prevents seizure-induced neuronal death (Caricasole et al., 2004; Busceti et al., 2007), but not ictal activity seizures (Busceti et al., 2007). These evidences suggest that the mechanisms for neuronal loss and seizure activity are separated, raising the possibility to use therapies targeting Wnt signaling components to protect against the neuronal loss induced by epilepsy.

## Wnt PATHWAY IN MAJOR MENTAL DISORDERS

In the last years an increased amount of evidence has shown that components of Wnt pathways are involved in major psychiatric disorders. Deregulation of Wnt signaling has been implicated in bipolar disorder (Klein and Melton, 1996; Grimes and Jope, 2001; Gould and Manji, 2002), in autism (Moon et al., 2004; De Ferrari and Moon, 2006), and schizophrenia (Miyaoka et al., 1999; Yang et al., 2003; Ftouh et al., 2005; De Ferrari and Moon, 2006; Inestrosa et al., 2012), among others. Bipolar disorder is a mental disorder characterized by episodes of depression interweaved by mania (Gould and Manji, 2002) but also for other misbalances such as sleep and circadian rhythm disturbances (Kaladchibachi et al., 2007; Ahnaou and Drinkenburg, 2011). At the molecular level, several evidences have converged to show that a dysregulation of GSK-3β cascade is involved in bipolar disorder (Klein and Melton, 1996; Grimes and Jope, 2001; Gould and Manji, 2002; Valvezan and Klein, 2012). It has been shown that mice with constitutively activated GSK-3β exhibit the same disturbances in sleep-wake cycle, locomotor activity, and body temperature, all major features that also can be found in bipolar patients (Tilleman et al., 2002; Prickaerts et al., 2006; Ahnaou and Drinkenburg, 2011). Lithium has been widely used as a treatment for mood disorders. Although GSK-3 seems to be the most plausible target for lithium (Valvezan and Klein, 2012), it has not been confirmed yet that GSK-3 is necessary for the action of lithium, since GSK-3 may activate several downstream mechanisms by which exerts its effects, such as Wnt and Akt signaling pathways (Valvezan and Klein, 2012). An evidence that link lithium effect with Wnt signaling is the fact that lithium stimulates neurogenesis (Samuels and Hen, 2011). Lithium, through GSK-3β inhibition, activates the canonical Wnt pathway to induce neurogenesis in the adult hippocampus (Lie et al., 2005). Moreover, transgenic mice overexpressing β-catenin shows the same behavior than lithium-treated animals suggesting that behavioral effects of lithium may be mediated through the increment of β-catenin (Gould et al., 2007). Additional studies are required to investigate the involvement of Wnt in lithium-mediated neurogenesis and behavior.

Recently, lithium has been also used to treat Ts65Dn mice, a model of Down syndrome. It has been shown that these animals exhibit several dysfunctions, such as synaptic failures and a significant reduced neurogenesis (Contestabile et al., 2013). Lithium, through the activation of canonical Wnt pathway, recovered the adult neurogenesis in these mice by stimulating the proliferation of neural precursor cells and increasing the number of newborn cells turning into mature neurons in the DG (Chen et al., 2000; Wexler et al., 2008; Contestabile et al., 2013). More importantly, the consequence of this treatment is the improvement of synaptic plasticity and cognitive functions dependent on hippocampal plasticity (Contestabile et al., 2013). It has been shown that Down syndrome has an excessive GABA inhibition that could exert a negative effect on the neurogenesis during embryonic and postnatal development, and on the excitatory neurotransmission, altogether leading to performance impairment (Kleschevnikov et al., 2004; Fernandez et al., 2007). Whether activation of Wnt pathway by lithium helps to partially restore the imbalance in the neurotransmission in this model, is a matter of further investigations. However, if so it suggests a possible therapeutic treatment to improve cognitive deficits in this disorder.

Wnt components such as GSK-3β have been also involved in psychotic and hyperactivity behavior, also referred as dopamine/serotonin-associated behaviors (Beaulieu et al., 2009). Several studies have converged evidence for an involvement of Akt and GSK-3 pathway in dopamine-related behavior and in the mechanism of action of some psychoactive drugs (Beaulieu et al., 2004; Beaulieu et al., 2005). It has been shown that overactivation of dopamine receptor-2 (D_2_R) causes hyperactivity through up-regulation of GSK-3β. The fact that the activation of Wnt pathway causes the inactivation of GSK3β could provide a window where to explore the potential usage of Wnt analogs as a treatment to improve the physical manifestation of psychosis and hyperactivity disorders.

Similar approaches directed to regulate GSK-3β can be found in the serotonin-related behavior. One of the consequences of the reduction of brain serotonin levels is the pronounced increment of GSK-3β and several emotional abnormalities such as anxiety and aggression in rodents (Beaulieu et al., 2008). Treating this animals with a specific GSK-3β inhibitor or breeding this animal with an animal deficient in GSK-3β, completely rescues the behavioral abnormalities associated with the lack of serotonin (Lucki et al., 2001; Weisstaub et al., 2006). This strongly suggests that GSK-3β signaling mediate behavioral effects of serotonin. Moreover, it has been shown that the typical antipsychotic drug haloperidol and the two atypicals risperidone and clozapine increase the protein level of β-catenin, Dvl-3, Axin, GSK-3α/β, and Wnt5a in prefrontal cortex, and β-catenin, GSK-3α/β, and Dvl-3 in the dopamine centers of the ventral midbrain (Alimohamad et al., 2005a,b; Sutton et al., 2007). The antipsychotics drugs do not have any effect on Wnt1 and Wnt3a, on Fz, or JNK or c-JNK (Sutton et al., 2007). These treatments cause an increment of β-catenin in the cytoplasm and nucleus and involve TCF-mediated gene transcription, suggesting that an abnormal expression of these proteins could underlie the serotonin-mediated behavior.

Recently, important evidences have related depression to Wnt signaling cascades (Duman and Voleti, 2012). It has been shown that a decreased BDNF expression underlies the stress and depression and, conversely, the use of antidepressants causes enhanced BDNF expression that could mediate the beneficial effects in the hippocampus and prefrontal cortex (Duman and Monteggia, 2006; Krishnan and Nestler, 2008). In fact, BDNF is sufficient to produce antidepressant behavioral effect; however deletion of BDNF is not enough to induce depression (Duman and Monteggia, 2006; Schmidt and Duman, 2007). BDNF has been recently described as a target of Wnt signaling (Yi et al., 2012). On the other hand, it remains unresolved whether the increase in BDNF levels is the result of Wnt signaling activation in these brain regions. However, other evidences could sustain this possibility. Also, it has been reported that GSK3 is required for the rapid antidepressant actions of ketamine (Beurel et al., 2011). Ketamine increases GSK3 phosphorylation in the mouse hippocampus and cerebral cortex (Beurel et al., 2011), while microarray studies show that the use of antidepressants differentially regulates the expression of Wnts, Fz receptors and Dvl in hippocampus, as well as Tcf/Lef, demonstrating the relevancy of the Wnt signaling components in the antidepressant effects (Okamoto et al., 2010; Duman and Voleti, 2012). Still more studies are required to elucidate the causes and potential beneficial effects of Wnt signaling on depression and related mood disorders.

As we previously mentioned, it has been shown that β-catenin regulates the expression of a novel group of genes in the thalamus (Wisniewska et al., 2012). However, it is still not clear whether variations in the level of β-catenin affect the expression of the genes that encode voltage gated Ca^2^^+^ channels (VGCCs) and neurotransmitter receptors, shaping neuronal excitability *in vivo*. If so, then inappropriate activity of β-catenin might affect the proper signal transmission in thalamocortical circuits. Thalamocortical desynchronization underlies absence epilepsy (Hughes, 2009). Specifically, the T-type voltage-gated channel Cav3.1 has been proposed to be implicated in absence seizures (Barclay et al., 2001; Liu et al., 2007; Ernst et al., 2009). Moreover, anticonvulsant drugs target voltage-gated channels (Errington et al., 2005). In the opposite, schizophrenia has also been associated with thalamic dysfunction (Harms et al., 2007; Smith et al., 2011; Marenco et al., 2012), while a group of synaptic genes involved in excitability have been found to be associated with the risk of schizophrenia (Lips et al., 2012). These results suggest a possible role for β-catenin-dependent gene expression in thalamic pathologies.

## Wnt-RELATED DEVELOPMENTAL ABNORMALITIES IN NEUROLOGICAL DISEASES

Literature has been recently populated with evidences indicating that changes during development could account for neurological disease vulnerability in the adulthood (Penzes et al., 2011; Penzes et al., 2013). Genetic mutations or environmental insults that perturb a normal developmental progression could constitute an early signature of developmental disorders. Nevertheless, disturbances that affect synapse morphogenesis and/or function during a specific developmental time or in a particular brain region, also may determine the consequent neuropathological symptom, such as neuronal circuit alterations and ultimately cognitive and behavioral symptoms. Mutations associated to initial cognitive disabilities may occur in molecules that play an essential role in regulating brain synapse formation and plasticity. Given the relevancy that Wnt signaling pathways have in these processes, we hypothesized that failures in establishing spines dendritic network could be caused by failures in the Wnt signaling pathways during development.

Some recent findings have provided important evidences to support this idea. Individuals with autism, SZ and AD show alterations in the density of dendritic spines in cortical pyramidal cells (Penzes et al., 2011) and also exhibit functional and structural changes in the inhibitory circuits (Oblak et al., 2010, 2011; Chattopadhyaya and Cristo, 2012). SZ patients show a profound reduction in spines density in prefrontal cortex and in primary auditory cortex, and also a reduction in the spines density and size in the hippocampal CA3 region In fact, the disrupted in schizophrenia (DISC1) gene is highly abundant in spines and it has also been associated to other psychiatric disorders (Schumacher et al., 2009). Indeed, DISC1 is known to interact with several well-established regulators of dendritic spine morphogenesis (Millar et al., 2003). Kalirin-7 via activation of a common Wnt pathway downstream effector Rac1 was found to directly regulate the effects of DISC1 on the spines morphology (Hayashi-Takagi et al., 2010). Kalirin- knockout mice remarkably show low spines density and behavioral schizophrenia-related behaviors at an age in mice that match to human adolescence, when the symptoms in humans emerges.

## CONCLUDING REMARKS

Emerging evidences suggest a role for Wnt pathway in synaptic maintenance and function of the adult brain. Activation of Wnt pathway has an effect in both basal and evoked activity. As we have discussed in this review, Wnt leads to rapid modification of the pre-synaptic compartment, regulating the clustering of synaptic proteins and the recycling of synaptic vesicles, while at the post-synaptic compartment Wnt modulates the assembly of the post-synaptic apparatus and the dendritic spine morphogenesis. Recent evidences indicate that activation of Wnt pathway modulates the efficacy of the excitatory and inhibitory synaptic transmission through divergent mechanisms. Moreover, activation of Wnt pathway enhances synaptic plasticity and the cognitive function of the adult brain. *In vivo* studies have shown that Wnt pathway is involved in both hippocampal- and amygdala-dependent memory. Interestingly, these studies have evidenced that endogenous Wnt activity is required for the normal functioning of neuronal circuits and that the inhibition of Wnt pathway can cause synaptic plasticity impairments and memory deficits. Moreover, the inhibition of Wnt pathway also causes a reduction in the basal spontaneous activity, which suggests that Wnt participates in the establishment of mature neuronal networks. Deregulation of Wnt pathway has been implicated in neurological pathologies, including AD, SZ, and mood disorders. Particularly, Wnt activator may be therapeutically relevant in AD since it has been shown activation of Wnt displays a neuroprotective effect and resolves the synaptic and cognitive impairment in mouse model of AD. In summary, the evidences discussed here show that Wnt pathway plays a key role in the synaptic function of mature nervous system, constituting a promising therapeutic target for the treatment of certain neurological diseases.

## Conflict of Interest Statement

The authors declare that the research was conducted in the absence of any commercial or financial relationships that could be construed as a potential conflict of interest.
